# Association of hoarding case identification and animal protection programs to socioeconomic indicators in a major metropolitan area of Brazil

**DOI:** 10.3389/fvets.2022.872777

**Published:** 2022-10-03

**Authors:** Raphael Rolim de Moura, Wagner Antonio Chiba de Castro, João Henrique Farinhas, Graziela Ribeiro da Cunha, Martha Maria de Oliveira Pegoraro, Louise Bach Kmetiuk, Andrea Pires dos Santos, Alexander Welker Biondo

**Affiliations:** ^1^Department of Veterinary Medicine, Federal University of Paraná State, Curitiba, Paraná, Brazil; ^2^Coordination of the Metropolitan Region of Curitiba, Secretariat of Urban Development and Public Works of Paraná State, Curitiba, Paraná, Brazil; ^3^Latin-American Institute of Life and Nature Sciences, Federal University for Latin American Integration (UNILA), Foz do Iguaçu, Paraná, Brazil; ^4^Paraná State Secretary of Health, Curitiba, Paraná, Brazil; ^5^Department of Comparative Pathobiology, Purdue University, West Lafayette, IN, United States

**Keywords:** animals hoarders, hoarding behavior, pet welfare, One Health, human health, animal health

## Abstract

The present study assessed the identification of animal and object hoarding disorder cases by contact and mapping and the presence of animal protection programs in association with seven social–economic indicators of the metropolitan area of the ninth-biggest metropolitan area of Brazil. City Secretaries of Health and Environment provided demographic information and responded to a questionnaire. Overall, a very high level of hoarding case identification per municipality was associated with a higher Human Development Index, population, density, and income and related to distance from Curitiba, the capital of Parana State. Low and very low levels of hoarding case identification were related to greater area, higher Social Vulnerability Index (SVI), inequality, illiteracy, and rural areas. Very high identification level of animal protection programs was also associated with higher HDI, density and population, urban area, and high income, and geographical area. Similarly, low and very low levels of animal protection programs identification were major explained by low income, illiteracy, and distance related to higher population, urbanization, and higher HDI. In summary, better identification of hoarding cases and animal protection programs have shown an association with better socioeconomic indicators and higher population, density, and urban area. Whether municipalities with better human socioeconomic indicators may stimulate society's demands for identification of cases of individuals with hoarding disorder and animal programs should be further established. Regardless, animal health and welfare have been associated with improving human quality of life in a major Brazilian metropolitan area.

## Introduction

Hoarding disorder is characterized by progressive accumulation of objects and/or animals, poor hygienic conditions, and refusal of object disposal and animal adoption (1). Unsanitary conditions due to garbage accumulation, blockage of household areas, and disease transmission lead to impairment of human, animal, and environmental health (1). Hoarding disorder has been classified as a psychiatric obsessive-compulsive disorder, and animal hoarding has already been reported in several countries, including the USA (2), Australia (3), Spain (4), Canada (5), UK (6), Italy (7), Singapore (8), and Brazil (9). The presence of animal/object hoarding cases in Curitiba, Brazil, was recently estimated at 1 per 15,500 (6.45 cases per 100,000) inhabitants (9), which was seen more frequently in women, who were more prone to animal hoarding behavior. The same study also reported that risk factors associated with hoarder behavior have included the proliferation of pests and vectors, risk of fire (e.g., bare wiring or no available electricity leading to the use of candles), and landslips (e.g., object stacking and accumulation falling over persons and animals) (10). Protection programs and population management of companion animals have been increasingly established by non- and governmental agencies worldwide, mostly performing neutering-spaying services, responsible guardianship programs, animal cruelty surveillance, and adoption of stray, abandoned, and relinquished pets (11). Such programs have been historically established due to society's demands for control, prevention, and monitoring of pet abandonment, cruelty, and overpopulation, which is particularly aggravated in developing countries like Latin America (12).

The One Health approach, defined as a holistic assessment of human, animal, and environmental health, has reportedly been used to better understand zoonotic diseases in hoarding disorder cases, as previously shown by our research group for leptospirosis and toxoplasmosis (13–15). In addition, our group has also recently proposed and applied a One Health Index (OHI) to comprehensively measure human, animal, and environmental health indicators in Curitiba as a tool for the worldwide assessment of major metropolitan areas (16).

Although animal protection programs have improved in major metropolitan areas, no study has been performed to assess whether such human–animal health associations may be present at the municipality level. Moreover, hoarding case identification and mapping (with address locations showing hoarding monitoring), and animal protection programs may serve as consistent parameters for comparative animal health evaluation. Although geospatial locations herein were not fully available or reliable, a previous study of our research group has already shown that hoarding clusters were present at Curitiba, particularly in the downtown area (9), and such mapping represented a more effective approach to control and prevent hoarding hotspots and their impact on public health. Accordingly, the present study has aimed to assess case identification and mapping of individuals with hoarding disorder (object and/or animal) and animal protection programs in Curitiba, the ninth-biggest metropolitan area of Brazil with 29 municipalities and around 3.2 million inhabitants, along with their potential association with human social–economic indicators.

## Methods

City Secretaries of Health and Environment were officially contacted, and questionnaires were directly given to all 29 municipalities of the Curitiba Metropolitan Region (Supplementary Figure 1). Questionnaires, included questions on protection programs for domestic animals, objects, and/or animal hoarding, estimates of individuals with hoarding disorder, and which municipality section was responsible for hoarding disorder problems at the time (Supplementary material 1; Tables 1, 2). In addition, data on geographical, economic, and social indicators for each municipality were obtained (Table 3).

**Table 1:** Indicators and levels of hoarding cases identification out of 29 cities included in the metropolitan area of Curitiba, Parana State, Brazil^*^.

**City**	**Contact of individuals with animal hoarding disorder**	**Mapping of individuals with animal hoarding disorder**	**Contact of individuals with object hoarding disorder**	**Mapping of individuals with object hoarding disorder**	**Level of hoarding cases identification**
1. Adrianópolis	No	Yes	No	No	Very low
2. Agudos do Sul	Yes	Yes	Yes	No	Very low
3. Almirante Tamandaré	Yes	Yes	Yes	No	Average
4. Araucária	Yes	No	Yes	No	High
5. Balsa Nova	Yes	No	Yes	No	Very high
6. Bocaiuva do Sul	Yes	No	No	No	Low
7. Campina Grande do Sul	No	No	No	No	Average
8. Campo do Tenente	No	No	No	No	Very low
9. Campo Largo	Yes	Yes	Yes	No	High
10. Campo Magro	Yes	No	Yes	Yes	Average
11. Cerro Azul	Yes	No	Yes	Yes	Low
12. Colombo	Yes	No	Yes	No	High
13. Contenda	Yes	Yes	Yes	No	Average
14. Curitiba	Yes	Yes	Yes	Yes	Very high
15. Doutor Ulysses	Yes	No	Yes	No	Very low
16. Fazenda Rio Grande	Yes	No	Yes	No	Average
17. Itaperuçu	Yes	No	Yes	No	Very low
18. Lapa	Yes	No	Yes	No	High
19. Mandirituba	Yes	Yes	Yes	Yes	High
20. Piên	Yes	No	Yes	Yes	Low
21. Pinhais	Yes	Yes	Yes	Yes	Very high
22. Piraquara	Yes	Yes	Yes	Yes	Very high
23. Quatro Barras	Yes	No	Yes	No	Very high
24. Quitandinha	No	No	No	No	Low
25. Rio Branco do Sul	Yes	No	Yes	No	High
26. Rio Negro	Yes	No	Yes	No	Very low
27. São José dos Pinhais	Yes	Yes	Yes	Yes	High
28. Tijucas do Sul	Yes	Yes	Yes	Yes	Low
29. Tunas do Paraná	No	Yes	No	No	Very low

^*^Values as described in methods.

**Table 2:** Indicators and levels of animal protection identification out of 29 cities included in the metropolitan area of Curitiba, Parana State, Brazil^*^.

**City**	**Animal protection** **plan**	**Animal protection program**	**Microchip identification**	**Neutering program**	**Responsible ownership program**	**Animal cruelty service**	**Other animal programs**	**Level of animal protection identification**
1. Adrianópolis	No	No	No	No	No	No	No	Very low
2. Agudos do Sul	No	No	No	No	No	No	No	Very low
3. Almirante Tamandaré	Yes	Yes	No	Yes	No	Yes	No	Average
4. Araucária	Yes	Yes	Yes	Yes	No	Yes	No	High
5. Balsa Nova	Yes	Yes	Yes	Yes	Yes	Yes	Yes	Very high
6. Bocaiuva do Sul	No	Yes	No	No	No	No	No	Low
7. Campina Grande do Sul	No	Yes	No	Yes	No	Yes	Yes	Average
8. Campo do Tenente	No	No	No	No	No	No	No	Very low
9. Campo Largo	Yes	Yes	Yes	Yes	No	Yes	No	High
10. Campo Magro	Yes	Yes	No	Yes	No	Yes	No	Average
11. Cerro Azul	No	Yes	No	No	No	Yes	No	Low
12. Colombo	Yes	Yes	Yes	Yes	Yes	Yes	No	High
13. Contenda	No	Yes	No	Yes	No	Yes	No	Average
14. Curitiba	Yes	Yes	Yes	Yes	Yes	Yes	Yes	Very high
15. Doutor Ulysses	No	No	No	No	No	No	No	Very low
16. Fazenda Rio Grande	Yes	Yes	No	No	Yes	No	No	Average
17. Itaperuçu	No	No	No	No	No	No	No	Very low
18. Lapa	Yes	Yes	Yes	Yes	Yes	Yes	No	High
19. Mandirituba	No	Yes	Yes	Yes	Yes	Yes	No	High
20. Piên	Yes	No	No	No	No	No	No	Low
21. Pinhais	Yes	Yes	Yes	Yes	Yes	Yes	Yes	Very high
22. Piraquara	Yes	Yes	Yes	Yes	Yes	Yes	Yes	Very high
23. Quatro Barras	Yes	Yes	Yes	Yes	Yes	Yes	Yes	Very high
24. Quitandinha	No	Yes	No	No	No	Yes	No	Low
25. Rio Branco do Sul	Yes	Yes	No	Yes	Yes	Yes	No	High
26. Rio Negro	No	No	No	No	No	No	No	Very low
27. São José dos Pinhais	Yes	Yes	Yes	Yes	Yes	Yes	No	High
28. Tijucas do Sul	No	Yes	No	No	No	Yes	No	Low
29. Tunas do Paraná	No	No	No	No	No	No	No	Very low

^*^Values as described in methods.

**Table 3:** Main geographical, economic, and social indicators of the 29 cities included in the metropolitan area of Curitiba, Parana State, Brazil.

**Municipalities**	**Population (hab.)**	**Area (km^2^)**	**Density (hab./km^2^)**	**% Urban Area**	**Income per capita**	**HDI**	**SVI**	Illiteracy rate*(%)	Low income**(%)	**Distance from the capital (km)**
1. Adrianópolis	6,376	1,349.3	4.73	32	440.79	0.667	0.403	18.36	50.03	127
2. Agudos do Sul	8,270	192.2	43.02	34	519.63	0.66	0.3	9.55	36.56	70
3. Almirante Tamandaré	103,204	194.7	529.94	95	646.02	0.699	0.337	6.51	18.33	15
4. Araucária	119,123	469.2	253.9	92	814.39	0.74	0.304	3.81	14.09	29
5. Balsa Nova	11,300	349	32.38	60	652.54	0.696	0.267	4.85	20.41	52
6. Bocaiuva do Sul	10,987	826.3	13.3	46	547.26	0.64	0.394	9.77	31.09	40
7. Campina Grande do Sul	38,769	539	71.93	82	671.29	0.718	0.317	6.39	20.06	31
8. Campo do Tenente	7,125	304.5	23.4	58	749.21	0.745	0.265	4.77	17.60	91
9. Campo Largo	112,377	1,249.4	89.94	83	567.04	0.701	0.339	6.60	21.83	29
10. Campo Magro	24,843	275.6	90.15	78	488.06	0.686	0.299	9.81	39.09	21
11. Cerro Azul	16,938	1,341.2	12.63	28	342.88	0.573	0.363	19.36	54.12	86
12. Colombo	212,967	197.4	1,079.08	95	682.85	0.733	0.311	4.97	16.29	20
13. Contenda	15,891	299	53.14	58	612.80	0.681	0.281	5.37	28.85	46
14. Curitiba	1,751,907	435.3	4,024.84	100	1,581.04	0.823	0.253	2.22	7.86	0
15. Doutor Ulysses	5,727	781.5	7.33	16	277.33	0.546	0.451	19.21	63.97	163
16. Fazenda Rio Grande	81,675	116.7	700.02	92	677.31	0.72	0.339	4.80	18.31	26
17. Itaperuçu	23,887	314.4	75.97	83	468.04	0.637	0.381	11.75	32.79	29
18. Lapa	44,932	2,093.8	21.46	60	608.60	0.706	0.289	6.03	30.68	69
19. Mandirituba	22,220	379.2	58.6	33	539.68	0.655	0.365	7.13	31.11	43
20. Piên	11,236	254.9	44.08	40	911.51	0.751	0.261	3.56	11.94	86
21. Pinhais	117,008	60.7	1,926.09	100	581.74	0.7	0.332	5.33	23.29	10
22. Piraquara	93,207	227	410.54	49	541.67	0.694	0.239	4.48	27.35	22
23. Quatro Barras	19,851	181.1	109.59	90	800.40	0.742	0.284	5.05	14.72	25
24. Quitandinha	17,089	447	38.23	28	452.08	0.68	0.31	7.64	40.99	67
25. Rio Branco do Sul	30,650	812.3	37.73	71	548.80	0.679	0.388	11.35	30.27	31
26. Rio Negro	31,274	603.2	51.84	82	709.13	0.76	0.224	3.75	22.53	110
27. São José dos Pinhais	264,210	946.4	279.16	89	846.93	0.758	0.266	3.60	12.32	14
28. Tijucas do Sul	14,537	672.2	21.63	15	547.62	0.636	0.275	9.31	32.57	63
29. Tunas do Paraná	6,256	668.5	9.36	44	431.27	0.611	0.447	19.74	51.59	83

^*^Illiteracy measured on 18 years old or older. ^**^Proportion of persons within the same household with family income equal to or below half of the minimum wage.

Although city officials thoroughly answered the questionnaire to identify hoarding behavior cases and animal protection programs from each municipality, hoarding disorder cases have been mostly associated with citizen complaints of unhealthy household conditions, while animal protection programs have been related to animal welfare services, performed in collective affirmative efforts. Thus, hoarding behavior case identification and animal protection program presence were explored into two distinct profiles, separately compared to ensure optimum analysis. Such profiles were obtained per municipality and analyzed according to socioeconomic indicators, one for each identification.

### Data analysis

The indicators were parameterized following a binary logic, in which “yes” responses were one and “no” responses were zero, as previously established (16).

The first profile (hoarding profile) was the sum of four indicators (“Contact of individuals with animal hoarding disorder”, “Mapping of individuals with animal hoarding disorder”, “Contact of individuals with object hoarding disorder,” and “Mapping of individuals with object hoarding disorder”) (Table 1).

The second profile (animal protection profile) was the sum of seven indicators (“Animal Protection Plan”, “Animal Protection Program”, “Microchip Identification”, “Neutering Program”, “Responsible Ownership Program”, “Animal Cruelty Service,” and “Other Animal Programs”). The Animal Protection Plan has been defined as a broad and officially approved city hall plan with strategies and resources, the Animal Protection Program as a part of a city plan with unfolded, extended, and ramified projects, campaigns, and activities, the Microchip Identification as a city service of pet microchipping application along with owner and pet data registration and available archive, the Neutering/Spaying Program as a city pet population management program, the Responsible Ownership Program as part of city annual activities and insertion on city public elementary schools, the Animal Cruelty Service as a program based on a free phone number (156) for anonymous citizen complaints and daily professional visits for animal cruelty inspection, and the Other Animal Programs as a group of other initiatives toward animal protection and well-being including pet adoption and fundraising events (Table 2). The profiles were rated on a qualitative scale according to the number of positive answers. For the hoarding profile, degrees were translated into 0 = very low, 1 = low, 2 = average, 3 = high, and 4 = very high. For the animal protection profile, degrees were translated into 0 = very low, 1 and 2 = low, 3 and 4 = average, 5 and 6 = high, and 7 = very high.

Although a unique indicator for human quality of life, the Human Development Index—Municipality (HDI-M) represented a calculation of a composite index from three dimensions, which included knowledge, long and healthy life, and decent living standard. On the other hand, the social vulnerability index (SVI) comprised a total of 16 social indicators within three domains and is used herein as an overall human health indicator. In short, the SVI categories and variables included:

1. Urban infrastructure

1.1 Percentage of persons living in homes with inadequate water supply and sewage.
1.2 Persons living in urban homes with inadequate garbage service.
1.3 Persons living in homes with inadequate per capita income.

2. Human Capital

2.1 Mortality up to 1 year of age.
2.2 Children from 0 to 5 years of age not attending school.
2.3 Persons aged six to 14 years who do not attend school.
2.4 Women aged 10–17 years who have children.
2.5 Mothers in charge of the homes, no complete elementary school and with a child under 15.
2.6 Illiteracy rate of persons aged 15 years or over.
2.7 Children living in homes with other residents with incomplete elementary school.
2.8 Persons aged 15–24 years out of school, unemployed and low per capita income.

3. Income and Work

3.1 Persons living in homes with low per capita income.
3.2 Unemployment rate of the population aged 18 or older.
3.3 Persons aged 18 or over without complete elementary education and with informal work.
3.4 Persons in homes with low per capita income and dependent on the elderly.
3.5 Activity rate of persons aged 10–14 years.

Although both HDI and SVI represent broad composite indices, presented by the Brazilian Institute of Geography and Statistics as final absolute numbers and consequently not allowing to pinpoint the value of each factor, assessing the relevance of such indices as associated risk factors may lead to a better understanding of animal hoarding.

A multivariate statistical approach with a canonical correspondence analysis (CCA) was applied to evaluate the relationship between the identification levels of hoardings and animal protection programs (from very low to very high) and the socioeconomic indicators in the logarithm scale (17) using the software PAST, version 4.0.9. The hoarding profile included four indicators, while the animal profile presented seven indicators (each question was one indicator). To facilitate the comprehension of such outcome data, the hoarding and animal profiles were adjusted to a five-degree scale, as described in the data analysis. Thus, such a method may be applied to any number of indicators.

A generalized linear model (GLM) was applied to explore the relationship between the two indexes, named hoarding and animal protection program identification. The Axis 1 scores resulting from the CCA of both indexes have confronted each other. This approach was used due to the nonparametric nature of the linear model residuals. The GLM was implemented in the R statistical environment, as previously established (18). In short, the canonical correspondence analysis aimed to elucidate the relationships between assemblies (in our case, municipality quality) and their environments by arranging the variables along with the axes. Due to the nature of the ordination method, there was no level of associated significance and other model parameters. The CCA axis values were provided for each profile (Supplementary material 2).

### Ethical considerations

This study was approved by the National Human Ethics Research Committee (Protocol number 3,166,749/2019) and the Ethics Committee on Animal Use (Protocol number 077/2015) through the Federal University of Paraná, Southern Brazil.

## Results

**Figure 1:**
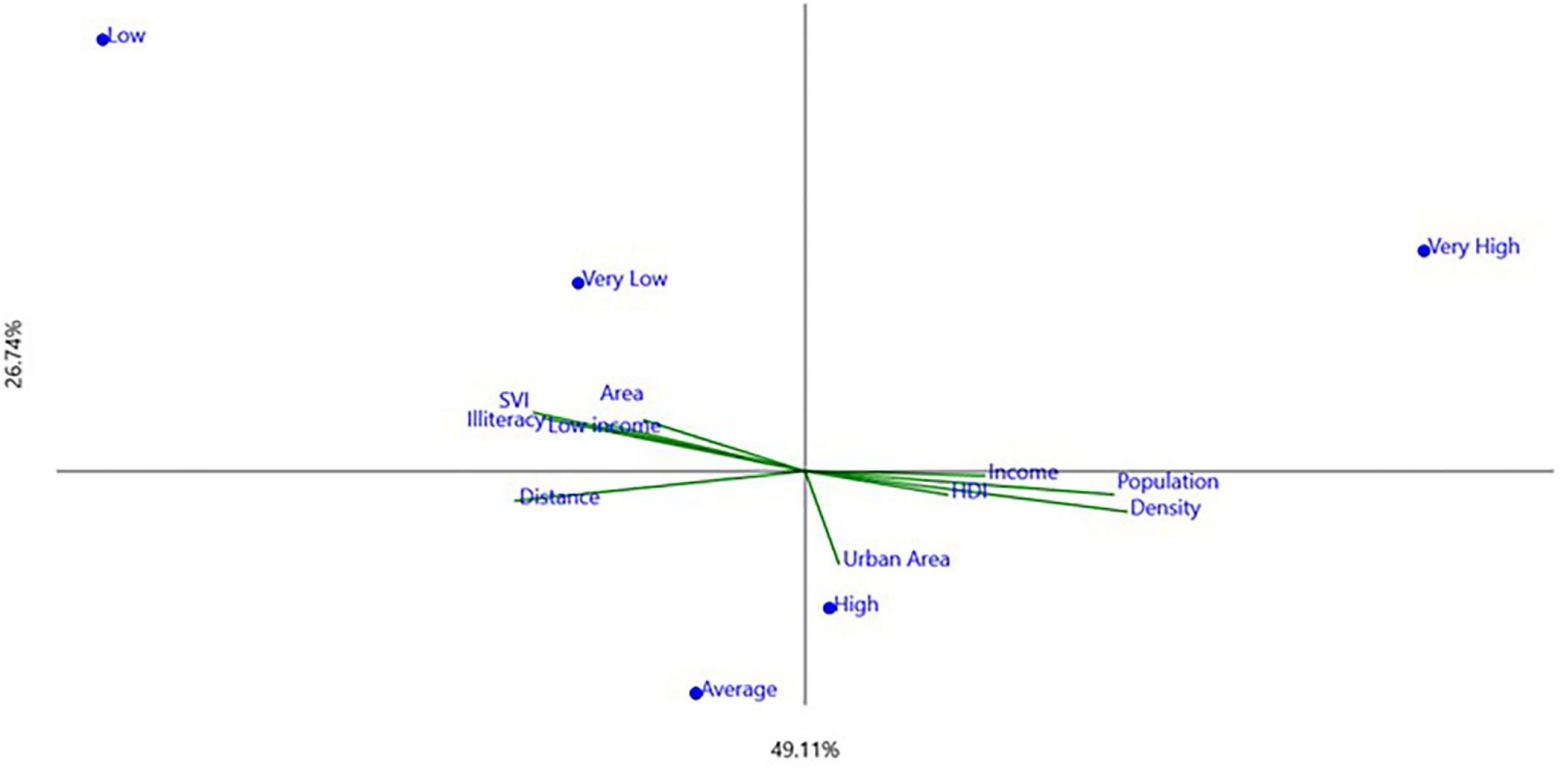
Graphic presentation of the canonical correspondence analysis exploring levels of hoarding identification (as dots, Table 1 data) and socioeconomic indicators (as vectors, Table 3 data). *X* (horizontal) and *Y* (vertical) axes explanations are presented (in percentage).

**Figure 2:**
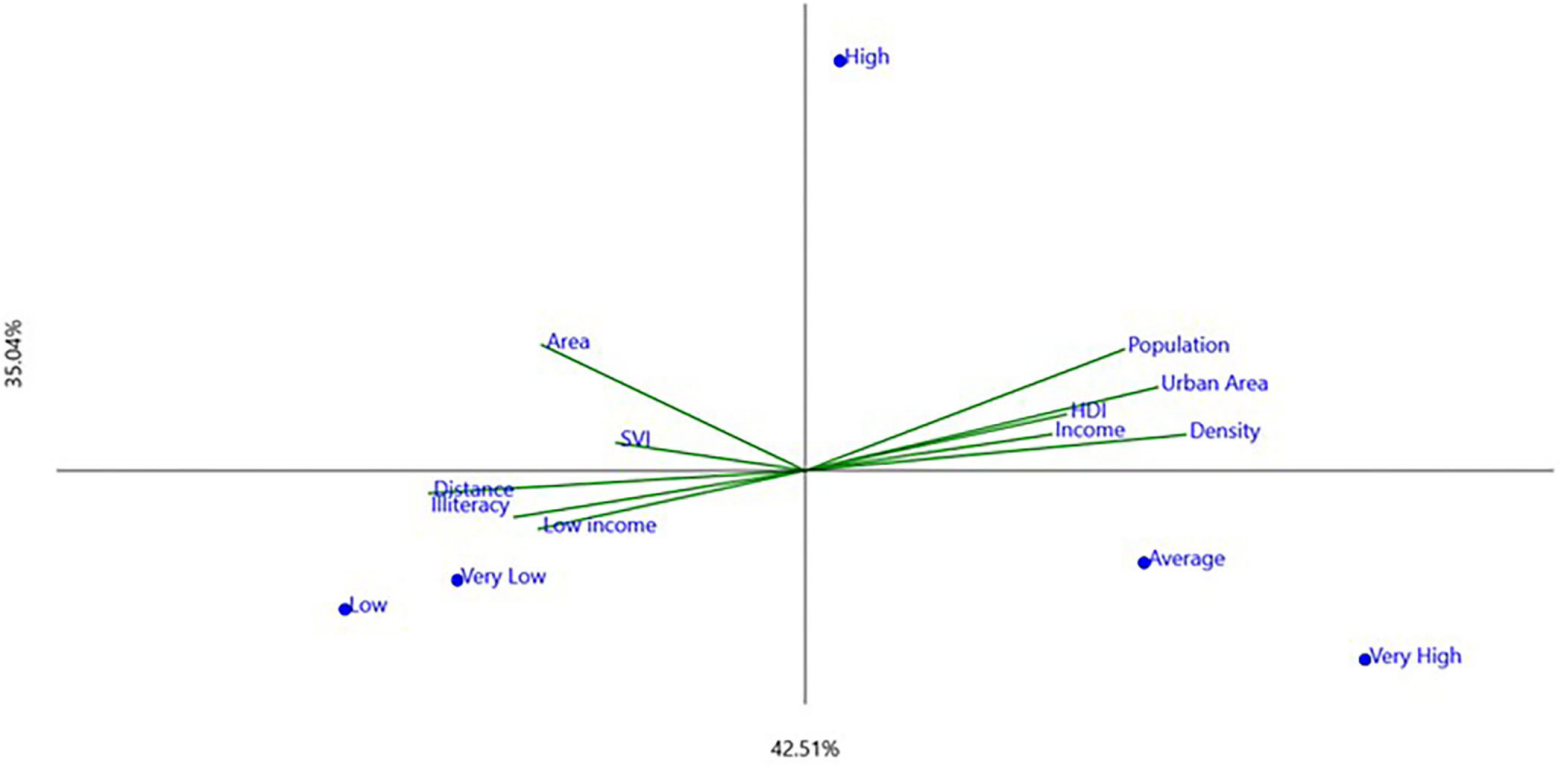
Graphic presentation of the canonical correspondence analysis exploring levels of animal protection programs (as dots, Table 2 data) and socioeconomic indicators (as vectors, Table 3 data). *X* (horizontal) and *Y* (vertical) axes explanations are presented (in percentage).

Results obtained from the questionnaires on the identification of hoarding cases and protection programs applied to the 29 municipalities were gathered and presented (Tables 1, 2). Each municipality's geographical, economic, and human development data were also presented as social–economic indicators (Table 3). Analyses of canonic correspondence (CCA), which are interrelated with qualitative and quantitative variables, have been performed and presented (Figures 1, 2).

Overall, 24/29 (82.8%) municipalities reported contact with animal hoarding disorder individuals, and 12/29 (41.8%) mapped these individuals; 23/29 (79.3%) had contact with object hoarding disorder individuals, and 09/29 (31.0%) mapped these individuals. In addition, 15/29 (51.7%) cities presented animal protection plans, 21/29 (72.4%) had animal protection programs, 11/29 (38.0%) performed microchipping identification, 16/29 (55.2%) conducted neutering/spaying programs, 11/29 (38.0%) directed responsible ownership programs, 19/29 (65.5%) had services against animal cruelty, and 6/29 (20.7%) referred other animal programs.

The CCA method robustness was quantified in percentage by each axis explanation. For the hoarding case identification profile, the x-axis explained 49.11% and the y-axis explained 26.74% of the association, meaning that municipalities with a very high level of hoarding cases identification were mostly explained as high urban areas located at a short distance from Curitiba (Figure 1). Municipalities with very high and high levels of hoarding cases were also related to higher Human Development Index (HDI), density, population, and income per capita. On the other hand, municipalities with low and very low levels of hoarding cases identification were explained by a higher distance from Curitiba, higher area, SVI, illiteracy, low income, population density, and urbanization.

For animal protection programs profile, the x-axis explained 42.51%, and the y-axis explained 35.04% of the association (Figure 2), with cartesian planes from the CCA plots explaining 77.55% and 75.85% of all data variability in hoarding and animal profiles, respectively, closely explaining the outcome data. Comparing the two profiles, the levels of animal protection program perception presented a significant and direct relationship with the levels of hoarding disorder perception (Table 4).

**Table 4:** Results of the generalized linear model between the hoarding perception index and the animal protection program perception index out of 29 cities belonging to the metropolitan area of Curitiba, Parana State, Brazil.

**Variable**	**Estimate**	**St. Error**	***t*-value**	***p*-value**
Intercept	0.25128	0.05586	4.499	< 0.001
Hoarding × Animal Protection program	0.46132	0.11786	3.914	< 0.001

## Discussion

Our study is the first to assess hoarding case identification and animal protection programs associated with socioeconomic indicators in a major metropolitan area worldwide. Fewer cases were identified in municipalities of larger geographic areas with small populations and density, which may be partially due to fewer occurrences and/or unnoticed cases. These areas were also more distant from the capital Curitiba and represented rural areas. The number of cases in such locations presented a higher correlation with low income and high illiteracy rates at different municipality levels, strongly indicating an association between low numbers of hoarding cases identification and low social indicators.

Although new approaches have been proposed for an optimum response to both human and animal problems (6), community poverty and illiteracy may impair hoarding identification, recognition, awareness, and prevention. Moreover, poor human and pet health outcomes in communities may be a consequence of multifactorial origin, including individual factors such as income, race/ethnicity, mental health, and structural factors such as over-policing, lack of healthcare, and housing discrimination (19). Thus, the findings herein have confirmed that improvement of animal health and welfare may require concomitant improvement of human health and welfare (as well as environmental health), as proposed by the One Health initiative (15).

Likewise, urbanized municipalities with higher HDI and lower SVI and higher population and population density have shown a higher correlation with higher hoarding case identification. As expected, the human–animal bond has reportedly improved pet health and welfare and positively impacted owners within a community (19). As hoarding disorder may simultaneously impact human, animal, and environmental health and welfare, One Health and One Welfare initiatives have already recognized such close interrelations, proposing holistic approaches through interdisciplinary teamwork (20). Not surprisingly, the intermediate classification “medium” was insufficient and not explained by the variables herein.

As animal and/or object hoarding disorder has been considered a major sanitary problem worldwide, no contact or mapping attempt by a given municipality was considered a negative indicator of human and animal health and welfare. Such an assumption was based on a recent study by our research group that found a high presence of animal and object hoarding disorder in Curitiba (9), which has been statistically associated with human and animal cruelty and unsanitary conditions (10). High identification of hoarders happened in municipalities with higher income and a composite of higher distance from the state capital and higher income. In other words, income was a direct determinant for the identification of hoarders. This sole finding has raised a series of questions including whether higher-income cities were more troubled by hoarding disorder, low-income cities were less likely to have hoarding persons, or rural hoarding was not identified due to poorer areas with low-income neighborhoods or just because presumably they were spaced farther apart so they were not aware of hoarding conditions. In addition, a previous study of our research group on hoarding behavior mapping of Curitiba has shown that hoarding cases were inversely associated with neighborhood income, suggesting that as neighborhood income decreased, the hoarding numbers increased (9). Despite findings that may appear contradictory, this previous study in Curitiba compared different income levels within city areas, showing that neighborhoods may not follow the pattern of surrounding metropolitan cities. Also, the early study was performed with actual identified hoarding cases, rather than the information herein, given by the city administration as a whole. Thus, the authors hypothesized that such discrepancy may indicate that, despite within city location of hoarders, more likely in lower-income neighborhoods, the overall city administration of higher-income cities may have more resources for hoarding specific protocols, personal, and services.

High identification of hoarders happened in municipalities with higher populations herein, corroborating our previous study that hoarding frequency was consistent with an increase in neighborhood population and human density, with a higher likelihood of complaints and identification in Curitiba (9). Such findings have reinforced the secretive nature of hoarding disorder, as accumulation may lead to household isolation and resident absence during a visit from city officials (9). As expected, a higher SVI, which indicates lower human health and association with lower-income cities, has shown a direct correlation with municipalities with low and very low hoarding identification. Average identification was predictably plotted near the center.

Inversely, municipalities with higher indexes of illiteracy have shown very low identification of cases of hoarding disorder. A higher SVI, which indicates lower human health, has also shown a direct correlation with municipalities with low and very low identification. Average identification, as expected, was plotted near the center. Although a few studies have compared animal protection to human socioeconomic indicators, self-reported awareness of dog ownership responsibilities was poor even in a well-educated Irish university community, with no difference between dog owners and non-dog owners (21).

Whether animal hoarders tend to live closer to the capital and bigger cities, merely that reporting systems may be better in an urban area, or the lower prevalence of animal hoarders in rural areas may be due to less animal welfare enforcement and fewer neighbors to make complaints remains unclear and should be further investigated. Although most societal indicators describe the literate (and not illiterate) percentage of a given population, illiteracy was used herein as the term presented by the Brazilian Institute of Geography and Statistics. While scoring higher on the HDI and lower on the SVI, animal hoarding associated with more densely populated areas and higher income levels may be due to better animal services, rather than more animal hoarding persons in such areas.

A previous study of our research group has found an overall ratio of 6.45 cases of hoarding disorder per 100,000 inhabitants in Curitiba, meaning that at least one case would be present in a neighboring city with 15,000 inhabitants (9). In such a scenario, cities with a lower population would likely have no animal hoarding persons. Surprisingly, out of cities under 15,000 people included herein, 6/9 (66.7%) presented hoarding cases, indicating a higher prevalence per population than previously reported. Although the presence of animal protection programs may be associated with increased identification of hoarding problems, these two profiles were evaluated separately and presented similar but distinct outcomes. The authors hypothesize that such differences may reflect the difficulties of accurate detection of hoarding disorder cases, which mostly rely on neighbor complaints followed by official visits and confirmation, requiring proper psychiatric evaluation, differential diagnosis, and continuous follow-up assessments.

Our research group has shown that a total of 113 out of 226 (50.0%) registered complaints were confirmed as hoarding cases by Curitiba city between 2013 and 2015, with 41/113 (36.3%) animal hoarding persons, 48/113 (42.5%) object hoarding persons, and 24/113 (21.2%) animal and object hoarding persons (45.4%) (4, 22). Considering these findings, the present study associated animal and object indicators as overlapping and separately to construct a composite indicator, which helped synthesize the hoarding case identification.

As a limitation, the presence of animal/object individuals with hoarding disorder in Curitiba was recently estimated at 1 case per 15,500 (6.45 cases per 100,000) inhabitants (9), and 9/29 municipalities had smaller populations below that at the time; they may have truly presented no hoarding cases. However, 6/9 municipalities had confirmed contact with individuals with animal hoarding disorder in the survey, suggesting that prevalence may have been previously underestimated. Despite the majority of cities herein reported animal (24/29, 82.8%) and object (23/29, 79.3%) hoarding contact, a future survey should be conducted to establish and compare the frequency of animal, object, or both hoarding disorders in the metropolitan area of Curitiba (9). In addition, as mentioned before, lower hoarding identification observed in municipalities of larger (probably rural) geographic areas and low population may be partially due to fewer and unnoticed cases.

Despite the present study having aimed to evaluate its interactions and associations, animal hoarding has been considered a challenging disorder due to several approach problems. Such difficulties may include hoarding recognition and rule out differentials such as animal protectors, recycling waste pickers, and collectors; once recognized, unwillingness to collaborate, refusal to receive home visits, undergo physical examinations and psychiatric appointments; and other concomitant health and mental disorders. Thus, the present study has been limited to the hoarding approach itself as an obsessive-compulsive disorder, characterized by the denial of own situation and reluctance to cooperate with city official health and social services.

The findings herein have indicated significant challenges faced by the current city official services, which may occur in other cities worldwide. Animal protection programs and hoarding disorder complaints have been conducted and supervised by two separate systems, which may work independently and without an appropriate crossover of information and referrals. In addition, at least three distinct city secretaries may visit and attend to hoarding cases, including the city secretary of health due to public health surveillance (feces, urine, rats, ticks, flies, and Dengue fever mosquitos); the city secretary of environment due to piles of useless and rotten material, along with sick pets and risk of animal cruelty; and the city secretary of social services, due to the vulnerable (mostly the elderly) and secluded household occupant. Thus, a “city hoarding taskforce” should be strongly recommended for effective control and prevention of hoarding cases and their impacts on public, animal, and environmental health, with encouragement on information sharing, multiprofessional meetings and visits, and interconnecting actions.

In summary, better identification of hoarding cases and the presence of animal protection programs have shown an association with favorable socioeconomic indicators (higher HDI, higher income per capita, lower SVI, and lower illiteracy), higher population density, and urban area. Whether municipalities with better human socioeconomic indicators may stimulate society's demands for better identification of hoarding cases and animal programs, or vice-versa, should be further established. Regardless, animal health and welfare have been associated with the improvement of human quality of life in a major Brazilian metropolitan area.

## Data availability statement

The original contributions presented in the study are included in the article/Supplementary material, further inquiries can be directed to the corresponding author.

## Ethics statement

The studies involving human participants were reviewed and approved by the National Human Ethics Research Committee 3.166.749/2019. The patients/participants provided their written informed consent to participate in this study. The animal study was reviewed and approved by the Ethics Committee on Animal Use (Protocol number 077/2015) through the Federal University of Paraná, Southern Brazil.

## Author contributions

RM, WC, and AB contributed to the conception and design of the study. RM, WC, LK, and AB wrote the first draft of the manuscript. RM, JF, WC, GC, MP, LK, AS, and AB wrote sections of the manuscript. All authors contributed to the manuscript revision and read and approved the submitted version.

## Funding

AB research was funded through the Araucaria Foundation of Parana (SUS2020111000010). This research has been supported by the Araucaria Foundation of Parana through a Grant proposal (CP 13/2019, Research Applied to One Health).

## Conflict of interest

The authors declare that the research was conducted in the absence of any commercial or financial relationships that could be construed as a potential conflict of interest.

## Publisher's note

All claims expressed in this article are solely those of the authors and do not necessarily represent those of their affiliated organizations, or those of the publisher, the editors and the reviewers. Any product that may be evaluated in this article, or claim that may be made by its manufacturer, is not guaranteed or endorsed by the publisher.

## Supplementary material

Supplementary Figure 1 Illustrative map of 29 municipalities in the Curitiba Metropolitan Region, as stated in 2022. Source: COMEC—Coordination of the Metropolitan Region of Curitiba.

Supplementary Material 1 Questionnaire applied to object and/or animal hoarding and protection programs of domestic animals.

Supplementary Material 2 CCA axis values.
