# Supplementary material for: Association of hoarding case identification and animal protection programs to socioeconomic indicators in a major metropolitan area of Brazil
Source: Front Vet Sci. 2022 Oct 3;9:872777. doi: 10.3389/fvets.2022.872777 (PMC9574217; doi:10.3389/fvets.2022.872777)
Supplement: Supplementary Figure 1 — Illustrative map of 29 municipalities in the Curitiba Metropolitan Region, as stated in 2022. Source: COMEC—Coordination of the Metropolitan Region of Curitiba. [file Data_Sheet_1.DOCX]

Supplementary Figure


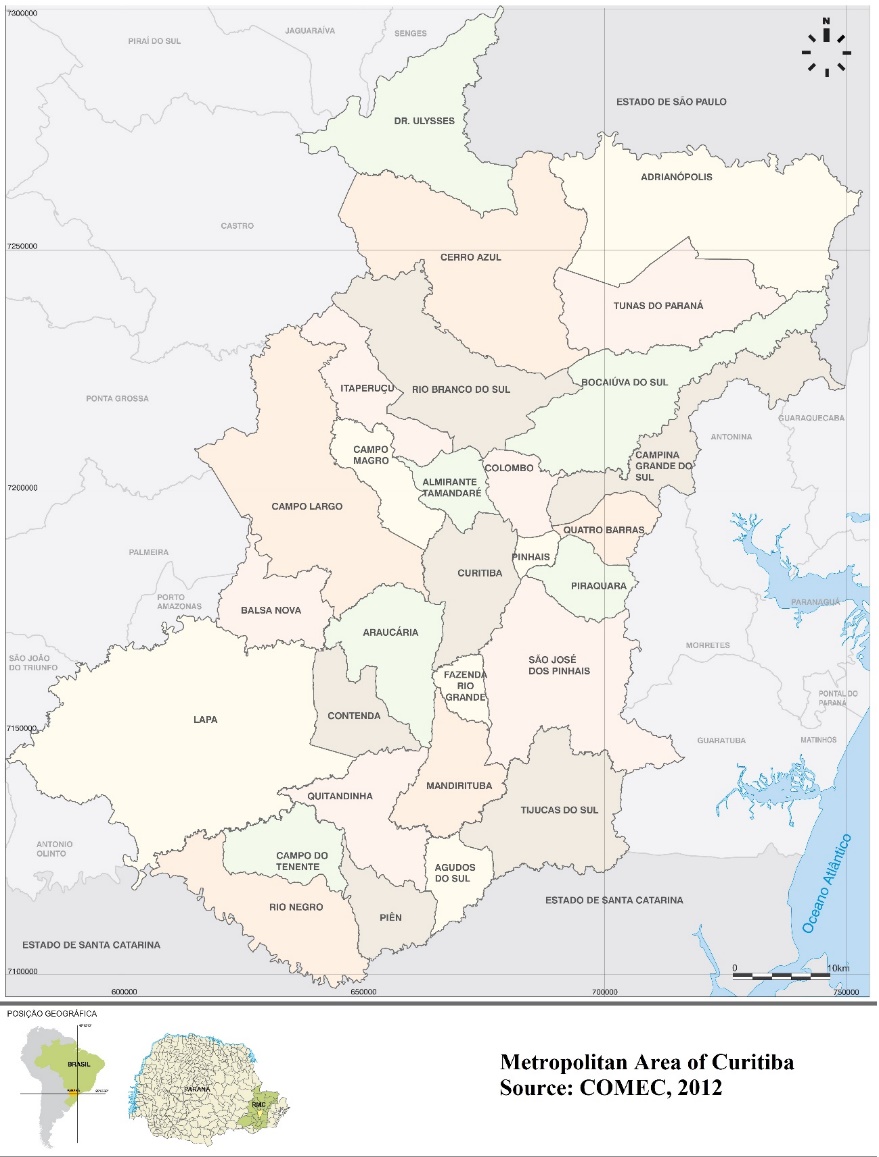


**Supplementary Figure 1.** Illustrative map of all 29 municipalities in the Curitiba Metropolitan Region, as stated in 2022. Source: COMEC - Coordination of the Metropolitan Region of Curitiba.
