# Supplementary material for: Association of hoarding case identification and animal protection programs to socioeconomic indicators in a major metropolitan area of Brazil
Source: Front Vet Sci. 2022 Oct 3;9:872777. doi: 10.3389/fvets.2022.872777 (PMC9574217; doi:10.3389/fvets.2022.872777)
Supplement: Supplementary Material 1 — Questionnaire applied to object and/or animal hoarding and protection programs of domestic animals. [file Data_Sheet_2.PDF]

Questionnaire of individuals with animal and/or object hoarding disorder in Curitiba metropolitan area, Brazil.

City: ________________________________

Name: ________________________________

Position: ________________________ E-mail: _______________________

1) Is there animal protection programs or other related programs destinated to individual with animal/object hoarding behavior?

   (   ) Yes.

   (   ) No.

2) If yes, which one?

   (   ) Animal identification (animal microchip)

   (   ) Neutering program (population management)

   (   ) Responsible ownership program

   (   ) Animal welfare control

   (   ) Other animal programs: _______________________________

   (   ) None.

3) Contact of individuals with animal hoarding behavior:

   (   )Yes.

   (   ) No.

   (   ) No information.

4) If yes, how many individuals with animal hoarding behavior are there in the city-mapping of individuals with animal hoarding behavior?

   ____

5) Contact of individuals with object hoarding behavior:

   (   ) Yes.

(   ) No.

(   ) No information.

6) If yes, how many individuals with object hoarding behavior are there in the city-mapping of individuals with object hoarding behavior

____
